# Brazilian Green Propolis and Essential Oil Residues Improve Metabolic Dysfunction by Modulating Key Hepatic Lipogenic Genes in High‐Fat Diet‐Fed Mice

**DOI:** 10.1111/1750-3841.71470

**Published:** 2026-09-16

**Authors:** Caroline Alves de Araújo, Giovana Dias Ramundo, Beatriz Teixeira dos Santos, Dafne Lopes Beserra Silva, Graziele Freitas de Bem, Dayane Teixeira Ognibene, Angela Castro Resende, Brenda Akemi Nagagata, Isabela Macedo Lopes Vasques‐Monteiro, Julio Beltrame Daleprane, Debora Baptista Pereira, Douglas Siqueira de Almeida Chaves, Cristiane Aguiar da Costa

**Affiliations:** ^1^ Department of Pharmacology, Institute of Biology Rio de Janeiro State University Rio de Janeiro Brazil; ^2^ Department of Basic and Experimental Nutrition, Institute of Nutrition Rio de Janeiro, State University Rio de Janeiro Brazil; ^3^ Laboratory of Pharmacognosy, Department of Pharmaceutical Science Federal Rural University of Rio de Janeiro Rio de Janeiro Brazil

**Keywords:** artepillin C, Brazilian green propolis, high‐fat diet, MASLD, obesity

## Abstract

Obesity is closely associated with metabolic dysfunction, leading to impaired glucose and lipid homeostasis and contributing to the development of metabolic dysfunction–associated liver disease (MASLD). Propolis is a bee‐derived natural product rich in bioactive compounds and widely recognized for its antioxidant, anti‐inflammatory, and immunomodulatory properties. This study evaluated the effects of Brazilian green propolis, a hydroalcoholic green propolis extract, and a residue‐derived extract obtained from the essential oil extraction process on high‐fat diet–induced MASLD in male C57BL/6 mice. Animals were allocated into five groups: control diet (10% fat), high‐fat (HF) diet (50% fat), HF supplemented with green propolis (HFGP, 2%), HF supplemented with green propolis extract (HFGPE, 2%), and HF supplemented with green propolis residue (HFGPR, 2%) for 12 weeks. Chemical analysis identified artepillin C as a major constituent in both extracts. Supplementation with Brazilian green propolis and its derivatives significantly reduced body weight gain, improved lipid profile and glycemic control, and exerted marked antioxidant effects. In addition, propolis supplementation downregulated the expression of key lipogenic genes and promoted a pronounced anti‐inflammatory response, resulting in a significant attenuation of hepatic steatosis. Collectively, these findings indicate that Brazilian green propolis effectively mitigates obesity‐induced metabolic disturbances and tissue damage. Notably, residue‐derived extracts from the essential oil extraction process represent a sustainable, cost‐effective, and promising therapeutic strategy for obesity and its associated metabolic complications.

## 1 Introduction

Obesity is closely linked to metabolic syndrome and contributes to disturbances in glucose and lipid metabolism, which may lead to the development of type 2 diabetes mellitus (T2DM) and metabolic dysfunction‐associated liver disease (MASLD) (Hateley et al. 2024).

MASLD is highly prevalent among individuals with obesity, affecting up to 96% of this population. Despite its high prevalence, only a proportion of these individuals, estimated between 7% and 35%, progress to metabolic dysfunction‐associated steatohepatitis (MASH), a more severe form of the disease characterized by hepatocellular injury, inflammation, and progressive lipid accumulation in the liver (Ooi et al. 2021).

MASH is characterized not only by excessive lipid accumulation in hepatocytes but also by hepatocellular injury and inflammatory infiltration. As the disease progresses, fibrosis develops due to the accumulation of extracellular matrix components and scar tissue within the hepatic parenchyma. In advanced stages, extensive fibrosis may progress to cirrhosis, a condition marked by severe architectural distortion of the liver and loss of function (Kalligeros et al. 2024; Park et al. 2024). Cirrhosis can lead to life‐threatening complications, including hepatic decompensation, hepatocellular carcinoma (HCC), and liver failure. With appropriate therapeutic interventions, including lifestyle modification and pharmacological strategies, regression of hepatic injury and even partial reversal of fibrosis may be achieved in selected cases (Park et al. 2024; Amernia et al. 2021).

Given the strong association between MASLD, obesity, and metabolic dysfunction, current management strategies primarily focus on lifestyle modifications. These typically include weight reduction through a balanced, healthy diet; reduced intake of ultra‐processed and fructose‐rich foods, particularly sugar‐sweetened beverages; and increased physical activity (Cusi et al. 2022; Kanwal et al. 2021). Notably, the incorporation of bioactive compounds derived from natural sources has emerged as a promising adjunct to these interventions, with growing evidence suggesting their potential to enhance metabolic health and mitigate key features of MASLD (Dungubat et al. 2025; Ciobârcă et al. 2025; C. Wang et al. 2025).

Propolis is a natural, safe, bee‐derived product composed of plant resins and bee secretions. It has been widely used in traditional medicine across various countries due to its broad spectrum of biological activities, including anti‐inflammatory, antioxidant, antimicrobial, anticancer, and immunomodulatory properties (Daleprane and Abdalla 2013; Pasupuleti et al. 2017; Okamura et al. 2022; Tsuda and Kumazawa 2021).

Although several plant species may contribute to the composition of Brazilian green propolis (GP), its primary botanical source is *Baccharis dracunculifolia* (Asteraceae), commonly known as *alecrim‐do‐campo*. This species belongs to the genus *Baccharis*, the largest within the subtribe Baccharidinae, which includes over 500 native species widely distributed across South America. *Baccharis dracunculifolia* is particularly prominent due to its abundance and chemical profile, serving as the main plant origin of Brazilian GP. This plant is notably rich in bioactive organic compounds, particularly artepillin C (APC), a phenolic compound associated with many of the pharmacological properties of GP (Washio et al. 2015; Baptista Pereira et al. 2024). This propolis has been reported to improve lipid metabolism (Nakajima et al. 2016), obesity, and insulin resistance in both humans (Fukuda et al. 2015) and animal models (Pasupuleti et al. 2017; Daleprane et al. 2012; Matsui et al. 2004).

However, studies remain scarce, particularly in the context of obesity, and investigations into the molecular mechanisms and signaling pathways involved in lipogenesis are still limited. Although the commercial production of Brazilian GP essential oil is currently limited, increasing interest in its biological Properties, including antimicrobial, antioxidant, anti‐inflammatory, and preservative activities, has expanded its potential applications in the pharmaceutical, cosmetic, and food industries. As these applications continue to develop, larger‐scale essential oil production may become economically feasible, generating significant quantities of solid residue. The recovery of bioactive compounds from this residue through hydroalcoholic extraction represents a sustainable valorization strategy that aligns with the principles of green chemistry and the circular bioeconomy by maximizing the utilization of propolis and minimizing industrial waste.

To date, no study has investigated the biological effects of Brazilian GP, a conventional hydroalcoholic extract, and a hydroalcoholic extract obtained from residues generated during essential oil extraction in the context of obesity‐induced metabolic dysfunction. Therefore, the present study was designed to evaluate three commercially relevant propolis‐derived preparations under identical dietary supplementation conditions, with particular emphasis on investigating whether the residue‐derived hydroalcoholic extract retains biologically relevant activity. Accordingly, the focus was placed on the biological performance of these preparations rather than on comparisons based on chemically standardized doses of specific bioactive constituents.

Using a high‐fat (HF) diet‐induced MASLD model in male C57BL/6 mice, we evaluated the effects of Brazilian GP, a conventional hydroalcoholic GP extract, and a hydroalcoholic extract derived from residues generated during the essential oil extraction process. To gain mechanistic insight, we investigated key pathways involved in lipogenesis, oxidative stress, and inflammation, which are central to the development and progression of MASLD.

## 2 Materials and Methods

### 2.1 Propolis and Extraction

Samples of Brazilian GP and their respective extracts were obtained in collaboration with Professor Douglas Siqueira de Almeida Chaves (Federal Rural University of Rio de Janeiro), as previously reported by Baptista Pereira et al. (2024). To obtain the hydroalcoholic extract of Brazilian green propolis (GPE), 500 g of raw propolis were mechanically ground and subjected to ultrasound‐assisted extraction using 70% ethanol (v/v) as the extraction solvent. The extraction procedure was performed in pulsed mode (pulse 1 at maximum power) for 5 min, then repeated three times to maximize recovery of bioactive compounds. The resulting extract was filtered, and the solvent was removed under reduced pressure using a rotary evaporator, yielding the crude dry hydroalcoholic extract.

To prepare the residue‐derived hydroalcoholic extract (green propolis residue [GPR]), 1000 g of Brazilian GP was hydrodistilled in a Clevenger‐type apparatus for 6 h to obtain the essential oil fraction. Following distillation, the remaining solid residue was collected, filtered, and dried. The dried residue was then subjected to ultrasound‐assisted extraction under the same conditions as those used for the raw propolis extract, using 70% ethanol (v/v) as the extraction solvent. After filtration, the solvent was removed under reduced pressure, resulting in the dry residue‐derived hydroalcoholic extract.

The extraction procedures yielded 20% (100 g dry extract from 500 g of raw Brazilian GP) for the hydroalcoholic extract (GPE) and 18.4% (184 g dry extract obtained from the residue generated after essential oil extraction of 1000 g of Brazilian GP) for the residue‐derived extract (GPR).

### 2.2 Total Phenolic and Flavonoid Content

The total phenolic content (TFen) for propolis extract (GP, GPE, and GPR) was determined by the Folin‐Ciocalteu method (Mara de Menezes Epifanio et al. 2020), and the standard curve was prepared using a solution of gallic acid in five different concentrations (0, 2, 5, 10, 20, 30 µg·mL^−1^). At these dilutions, 5.0 mL of water and 2.5 mL of Folin‐Ciocalteu reagent diluted (1:10 in distilled water) were added. Aliquot (0.5 mL) of the diluted sample of GP, GPE, and GPR in a methanolic solution (1.0 mg·mL^−1^) was transferred to an amber flask with a cap, and 5.0 mL of water and 2.5 mL of Folin‐Ciocalteu reagent (diluted 1:10 in water) were added. This mixture was stirred for 10 s and allowed to stand for 5 min. Aliquot (2.0 mL) of 4% sodium carbonate solution was added, the mixture was allowed to stand for 2 h, and the optical density was measured at 765 nm against a blank. The total phenolic contents were calculated on the basis of the calibration curve of gallic acid and expressed as gallic acid equivalents (GAE), in milligrams per gram of dry extract.

Total flavonoid content (TF) in propolis extract (GP, GPE, and GPR) was determined using the aluminum chloride colorimetric method (Mara de Menezes Epifanio et al. 2020). For this, 0.5 mL aliquots of each hydroalcoholic extract sample, in triplicate, were added to an equal volume of a 5% methanolic aluminum chloride (AlCl_3_) solution. After standing for 15 min, the absorbance was read at 420 nm. Total flavonoid content was determined using a standard quercetin curve at concentrations of 0, 5, 10, 20, 30, 40, and 50 µg·mL^−1^. Samples were independently analyzed in triplicate, and the total flavonoid content was expressed as mg quercetin equivalent per gram of dry extract.

### 2.3 In Vitro Antioxidant Activity

The percentage of antioxidant capacities (AC%) of each substance was assessed by 2,2‐diphenyl‐1‐picrylhydrazyl (DPPH) free radical assay (Mara de Menezes Epifanio et al. 2020). A total of 100 mL of methanolic DPPH solution (≈0.06 mmol.L^−1^) was obtained. This stock solution was prepared daily, used for the measurements, and kept in the dark at ambient temperature when not in use. Using this stock solution enables the construction of a calibration curve at 515 nm to calculate the DPPH concentration. Methanol was used as blank, and the samples used were the propolis extract (GP, GPE, and GPR) and the standard quercetin. The extracts were reacted with the stable DPPH radical in a methanol solution at five different concentrations, in triplicate. The reaction mixture consisted of 0.1 mL of sample to 3.9 mL of DPPH radical solution (0.06 mM in methanol). The color changes (from deep violet to light yellow) were measured at 515 nm after 55 min of reaction. The control solution was prepared by mixing 50% methanol (40 mL) and 70% acetone (40 mL). The scavenging activity (EC_50_) value indicates the amount of propolis extract required to reduce DPPH absorbance by 50%. The value can be determined graphically by plotting the absorbance against the used extract concentration or calculated by using the slope of the linear regression.

The ferric reducing antioxidant power (FRAP) reagent was freshly prepared from acetate buffer (300 mM acetate at pH 3.6), TPTZ solution (10 mM TPTZ in 40 mM HCl), and FeCl_3_ solution (20 mM) in the ratio 10:1:1 (v:v:v). The FRAP reagent (3 mL) was added to 100 µL of GP, GPE, and GPR in a test tube and incubated in a water bath at 37°C for 30 min. The color of the solution changed to dark blue, and its absorbance was measured at 593 nm. Fresh working solutions of known Fe (II) concentrations (FeSO_4_·7H_2_O) of (0‐2 mM) were used for the calibration curve (Mara de Menezes Epifanio et al. 2020).

### 2.4 High‐performance liquid chromatography coupled with diode‐array detection (HPLC‐DAD) Analysis

The chromatographic profiles of the extracts (GP, GPE, and GPR) were obtained using a Prominence liquid chromatograph (Shimadzu) equipped with two LC‐20AT series pumps, an SPD‐M20A photodiode‐array detector, and an SIL‐10A autosampler. Instrument control and data acquisition were performed using LCsolution software (Shimadzu). Analyses were carried out on a reversed‐phase C18 analytical column (250 mm × 4.6 mm, 5 µm particle size, Betasil, Thermo), maintained at 30°C, as previously reported by Baptista Pereira et al. (2024). The mobile phase consisted of water with 1% acetic acid and methanol, under gradient elution at a flow rate of 1.0 mL min^−^^1^. Compound identification was achieved by comparison of retention times and ultraviolet (UV) spectra with authentic standards, and quantification was performed using external calibration curves (Baptista Pereira et al. 2024). The relative abundance of the identified compounds was estimated based on the percentage of the chromatographic peak area. Therefore, the results provide a comparative assessment of the phytochemical profiles of the different propolis‐derived preparations and do not represent absolute concentrations of individual constituents.

### 2.5 Animals and Diet

All animal procedures were approved by the Ethics Committee for Care and Use of Experimental Animals (CEUA) of the Roberto Alcântara Gomes Institute of Biology/Rio de Janeiro State University (protocol CEUA No.025/2023, approved on June 14, 2023). The experimental procedures followed the conventional guidelines for animal experimentation (National Institutes of Health Publication, 8th edition). Mice were allocated to cages under standardized conditions with an appropriate temperature environment (21 ± 2°C) and a light‐controlled cycle (12 h of light and 12 h of darkness). We used 2‐month‐old male C57BL/6 mice obtained from the facilities of Rio de Janeiro State University. The animals were randomly divided into five nutritional groups fed for a 12‐week period, which included a control diet (control group: providing 10% of the energy from lipids, *n* = 15), an HF diet (HF group: providing 50% of the energy from lipids, *n* = 15), HF diet supplemented with GP 2% (HFGP, *n* = 15) (Okamura et al. 2022), HF diet supplemented with GP extract 2% (HFGPE, n = 15), and HF supplemented with GPR 2% (HFGPR, *n* = 15). The 2% supplementation refers to the dry weight of each respective material incorporated into the total diet weight (w/w): GP dry matter (GP), hydroalcoholic extract (GPE), or residue‐derived extract (GPR), all directly admixed into the diet before pelletization by the manufacturer (Rhoster, São Paulo, Brazil). All three materials were incorporated in their solid (dry) form following solvent evaporation under reduced pressure; no liquid extract was added to the diet. The total phenolic content of each material, determined by the Folin–Ciocalteu method and HPLC‐DAD, respectively, is reported in Tables 1 and 2, enabling cross‐group comparison of bioactive load.

**TABLE 1:** Total phenolic content (TFen), total flavonoids (TF), and antioxidant capacity (FRAP and DPPH) amounts for green propolis samples.

Samples	TFen^a^	TF^b^	FRAP^c^	DPPH^d^
GP	22.79 ± 0.020	31.51 ± 0.003	1482.86 ± 0.043	4.5 ± 0.005
GPE	19.45 ± 0.017^*^	37.12 ± 0.001^*^	807.15 ± 0.029^*^	4.37 ± 0.013^*^
GPR	13.04 ± 0.044^*+^	8.48 ± 0.042^*+^	801.12 ± 0.205^*+^	5.19 ± 0.040^*+^

*Note*: Values are expressed as mean ± standard deviation (*n* = 3). One‐way ANOVA.

Abbreviations: GP, green propolis; GPE, hydroalcoholic extract; GPR, residue extract.

^a^ mg GAE/100 mg.

^b^ mg QE/100 mg.

^c^ mmol Fe(II)/100 mg.

^d^ EC_50_ (ug/mL).

^*^Significantly different from the GP (*p* ≤ 0.05).

^+^Significantly different from the GPE (*p* ≤ 0.05).

**TABLE 2:** Retention time (min), maximum absorption—λ *max* (nm), and relative area (%) of phenolic compounds identified by HPLC‐DAD.

			Area (%)	
Compound	Tr (min)	λ max (nm)	GP/GPE	GPR
Chlorogenic acid	4.17	327	6.3	0.4
Caffeic acid	5.74	325	11.3	0.6
*p*‐Coumaric acid	8.46	310	17.2	5.0
Ferulic acid	8.19	240, 322	2.9	0.8
Rosmarinic acid	9.16	330	0.3	0.2
Pinobanksin	14.9	291	2.4	2.4
Kaempferol	11.02	267, 365	0.7	0.9
Drupanin	14.17	315	10.7	8.1
Kaempferide	22.54	266, 364	5.3	5.1
Artepillin C	25.6	314	22.3	23.5
Baccharin	22.5	315	8.2	15.1
Cromene	23.6	230	1.4	2.4

Abbreviations: GP, green propolis; GPE, green propolis extract; GPR, green propolis residue.

The three propolis‐derived materials (GP, GPE, and GPR) were incorporated into the experimental diets at the same concentration (2%, w/w). This formulation was established on a gravimetric basis to compare the biological effects of the different propolis‐derived products under identical dietary conditions. Therefore, the treatments were not standardized according to their ethanol‐soluble extractive content, total phenolic content, or individual bioactive constituents.

Accordingly, the present study was designed as a product‐based comparison rather than a comparison of chemically equivalent doses of bioactive compounds.

The animals had free access to HF or control diets during the 12‐week experimental period, manufactured according to the American Institute of Nutrition's recommendations (AIN‐93 M) (Reeves et al. 1993) and produced by Rhoster.

The animals were fasted overnight and euthanized under anesthesia (thiopental sodium, 70 mg/kg i.p.) after a 12‐week experimental period. Moreover, we collected blood samples via cardiac puncture into heparinized Eppendorf tubes, followed by plasma separation by centrifugation (120 × g for 15 min) at 4°C. The samples were then stored individually at −80°C until posterior analyses.

### 2.6 Food Intake, Body Weight, and Adipose Index

Food intake was recorded daily, and body weight was monitored weekly throughout the 12‐week experimental period, as previously described (Tavares et al. 2020; De Moraes Arnoso et al. 2025). Additionally, the adiposity index was calculated by dividing total fat mass, comprising intra‐abdominal and subcutaneous fat, by final body weight.

### 2.7 Glycemia and Plasma Assays

Blood glucose concentration was assessed by a glucometer (Accu‐Chek Active, Roche, Mannheim, Germany) in animals fasted for 6 h.

Plasma levels of total cholesterol (TC), triglycerides (TG), low‐density lipoprotein (LDL), very‐low‐density lipoprotein (VLDL), high‐density lipoprotein (HDL), alanine aminotransferase (ALT), and alkaline phosphatase (ALP) were evaluated using a kinetic assay (Bioclin, Belo Horizonte, MG, Brazil).

### 2.8 TC and TG Hepatic

Liver samples (50 mg) from each animal were stored at −80°C and subsequently homogenized in 1 mL of isopropanol using an ultrasonic processor. The resulting mixture was centrifuged at 2000 × g, and a 5‐µL aliquot of the supernatant was used to determine total cholesterol and triglyceride levels using a commercial assay kit on a semi‐automated biochemical analyzer (Bioclin, Belo Horizonte, Brazil).

### 2.9 Polymerase Chain Reaction Analyses

For this protocol, 50 mg of liver tissue previously stored at −80°C was used. Total RNA extraction was performed under RNase‐free conditions using the TRIzol reagent (15596026 ‐ Thermo Fisher Scientific, Inc., Waltham, MA, USA) according to the manufacturer's recommendations. The RNA was quantified using the BioDropTM spectrophotometer (Copyright 2025 Harvard Bioscience, Inc.). The cDNA was synthesized from 3 µg of total RNA using the High‐Capacity cDNA Reverse Transcription Kit (catalog No. 4368814; Applied Biosystems, Foster City, CA, USA). The mRNA expression of each gene was analyzed by real‐time reverse transcription quantitative polymerase chain reaction (RT‐qPCR), performed in duplicate for each sample using a Biosystems 7500 Applied Real‐Time PCR System (Applied BioSystems). The Gapdh (assay ID: Mm99999915) gene was used as an internal control. To calculate relative changes in gene expression from RT‐qPCR experiments, the 2−ΔΔCt method was used (Livak and Schmittgen 2001). The mRNA expression was measured using TaqMan Fast Advanced Master Mix (catalog No. 4352042; Applied Biosystems) according to the manufacturer's instructions. Primers used: *Il1b* (assay ID: Mm 00434228), *Acaca* (assay ID: Mm 01304258), *Ccl2* (assay ID: Mm 00441242), *Fabp4* (assay ID: Mm 00445878), *Srebp1* (assay ID: Mm00550338), *Ppargamma* (assay ID: Mm 00440940), *Cpt1a* (assay ID: Mm01231183), *Fgf21* (assay ID: Mm 07297622), and *Tnfa* (assay ID: Mm00443258).

### 2.10 Liver Oxidative Stress

Liver homogenates were used to assess oxidative damage by measuring lipid peroxidation levels, determined through the formation of malondialdehyde (MDA) using the thiobarbituric acid reactive substances (TBARS) assay (Draper and Hadley 1990).

The activities of antioxidant enzymes, including superoxide dismutase (SOD), catalase, and glutathione peroxidase (GPx), were measured in liver homogenates, following previously described protocols (Bannister and Calabrese 1987; Flohé and Günzler 1984; Aebi 1984). Total protein content in each sample was determined using the Bradford method.

### 2.11 Immunohistochemistry

Liver sections (5 µm) were deparaffinized, rehydrated, and incubated (10 min) with 0.3% H_2_O_2_ to block endogenous peroxidase. Subsequently, the cuts were incubated with 1% bovine serum albumin (BSA) diluted in phosphate‐buffered saline (PBS/BSA) to block nonspecific protein binding. In the next step, antigen retrieval occurred with trypsin (3%) administration diluted in distilled water for 10 min at 37°C or citrate buffer (at 60°C), following incubation with the primary antibody diluted 1:100 with 1% PBS/BSA overnight (4°C, in a humid atmosphere). We investigated the expression of the TIMP‐2 antibody (sc‐5539; Santa Cruz Biotechnology, CA, USA) and, for signal amplification, used a biotin‐streptavidin complex system (PK‐8800 Vectastain Universal quick kit; Vector Laboratories, Peterborough, UK). Finally, the positive immunoreactions were identified after incubation with 3,3′‐diaminobenzidine tetrachloride (K3466, DAB; Universal Dako Cytomation, Glostrup, Denmark). The sections were then counterstained with hematoxylin to visualize cell nuclei, and the slides were mounted and analyzed. Immunohistochemistry was evaluated by image analysis using the Image‐Pro Plus software.

### 2.12 Liver Morphology

Liver tissue samples were fixed, embedded in Paraplast Plus (Sigma‐Aldrich, St. Louis, MO, USA), sectioned at 5 µm, and stained with hematoxylin and eosin (H&E) for histological analysis under light microscopy. Images were acquired using an Olympus BX43 microscope equipped with a DP74 camera (Olympus, Tokyo, Japan). Hepatic fat density was estimated using a 36‐point test system, applying the formula Vv[fat] = Pp/PT, where Pp denotes the number of points that intersect fat droplets in 10 randomly selected fields per animal.

To assess liver fibrosis, tissue sections were stained with picrosirius red and visualized under light microscopy at 40× magnification (Olympus). For quantitative analysis, 15 fields per region per animal were selected, and collagen area (%) was measured using the Image‐Pro Plus software, with the results expressed as the average value per liver.

### 2.13 Statistical Analyses

Values are expressed as the mean ± standard error of the mean. Statistical significance was determined using one‐way analysis of variance, followed by Tukey's post hoc test. *p* values <0.05 were considered statistically significant (GraphPad Prism version 6.0 for Windows).

## 3 Results

### 3.1 Chemical Analysis

The total phenolic content (TFen) of the GP, hydroalcoholic extract (GPE), and the residue‐derived extract (GPR), calculated from the calibration curve (standard curve equation: *y* = 0.12497*x* + 0.12951; *R* ^2^ = 0.999), was 22.79 ± 0.020 mg GAE/g, 19.45 ± 0.017 mg·GAE/g, and 13.04 ± 0.044 mg·GAE/g, respectively. The total flavonoid (TF) content, determined using the calibration curve (*y* = 0.04078*x* + 0.06553; *R* ^2^ = 0.9841), was 31.51 ± 0.003 mg, 37.12 ± 0.001 mg, and 8.48 ± 0.042 mg of quercetin equivalents/g for GP, GPE, and GPR, respectively (Table 1).

Antioxidant capacity was assessed using the DPPH and FRAP assays. The DPPH EC_50_ values were calculated from the standard curve (*y* = 0.00107*x* + 0.0018; *R* ^2^ = 0.99961), with GPR exhibiting the highest EC_50_ value (5.19 ± 0.040 mg/mL). FRAP was expressed as mmol Fe(II) equivalents per gram of dry extract, and GP showed the highest FRAP value (1482.86 ± 0.043 mmol Fe(II)/g dry extract) (Table 1).

The statistical analysis showed that the TFen content differed significantly among the three preparations, with GP showing the highest value, followed by GPE and GPR (Table 1). In contrast, TF content was significantly higher in GPE than in both GP and GPR. Antioxidant capacity also differed significantly among the preparations. GP exhibited the highest FRAP, whereas GPR presented the highest DPPH EC_50_ value. Significant differences were observed among all three preparations for TFen, TF, FRAP, and DPPH (Table 1).

**FIGURE 1:**
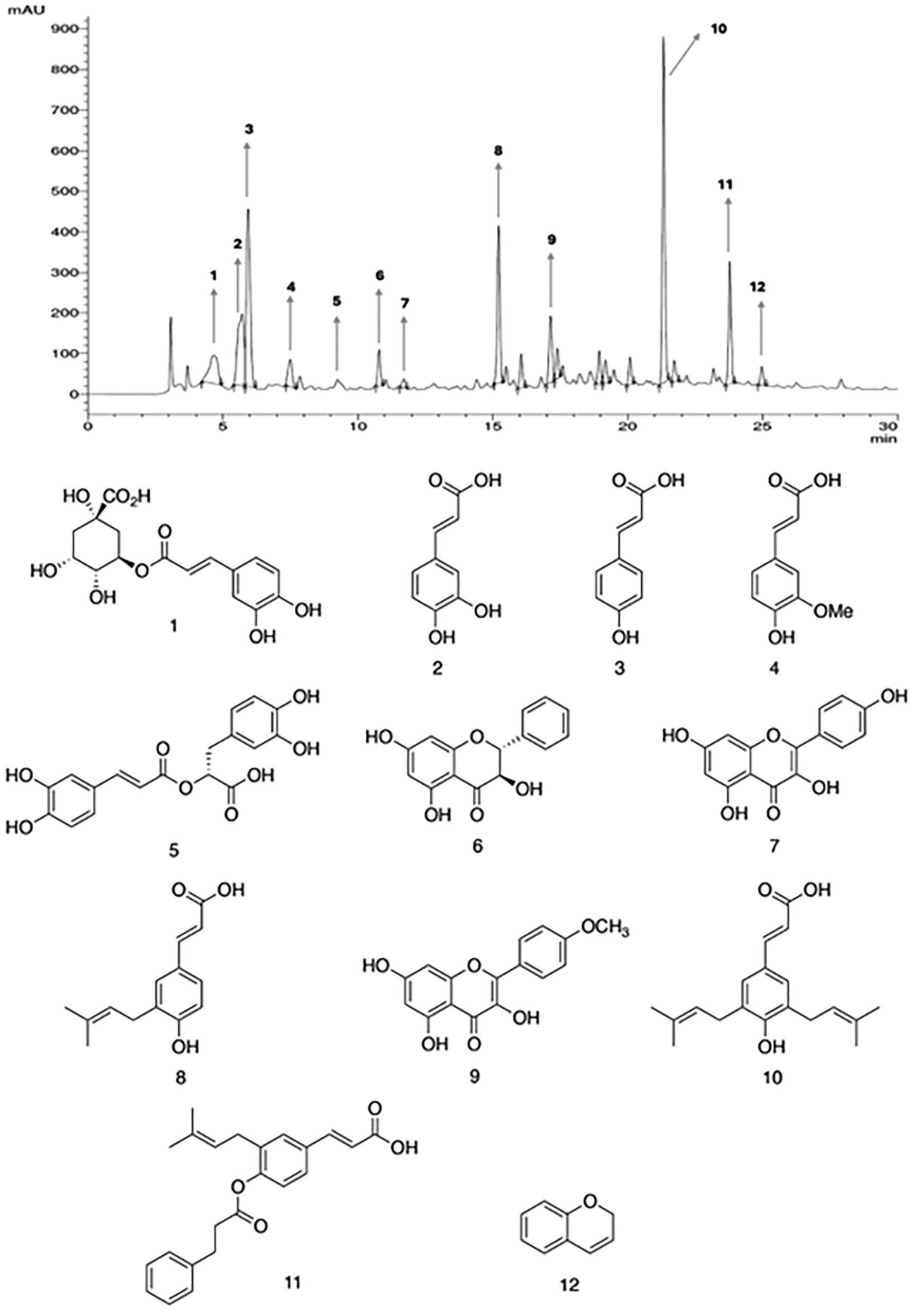
Chromatographic profiles (HPLC‐DAD base‐peak chromatogram) of green propolis extract and phenolic structures of compounds separated on a C18 column using an increasing methanol gradient in 0.1% aqueous formic acid. 1. Chlorogenic acid; 2. Caffeic acid; 3. *p*‐Coumaric acid; 4. Ferulic acid; 5. Rosmarinic acid; 6. Pinobanksin; 7. Kaempferol; 8. Drupanin; 9. Kaempferide; 10. Artepillin C; 11. Baccharin; 12. Cromene. The chromatographic data were expressed as relative peak area percentages obtained by HPLC‐DAD analysis. They should be interpreted as comparative compositional profiles rather than absolute quantitative measurements of individual compounds.

HPLC‐DAD analysis identified 12 compounds (Figure 1) based on their retention times and UV maxima (Table 2). The study showed a profile comprising phenolic acid derivatives, prenylated compounds, and glycosylated flavonoids. Notably, APC (3,5‐diprenyl‐4‐hydroxycinnamic acid) is a recognized biomarker for GP from southeastern Brazil. Our results showed that artepillin C was the major compound in GPE (22.3%) and GPR (23.5%), followed by *p*‐coumaric acid (17.2%), caffeic acid (11.3%), and drupanin (10.7%) in GP and GPE, and by baccharin (15.1%) in GPR.

### 3.2 Body Weight, Adiposity Index, Food Intake, and Energy Intake

**FIGURE 2:**
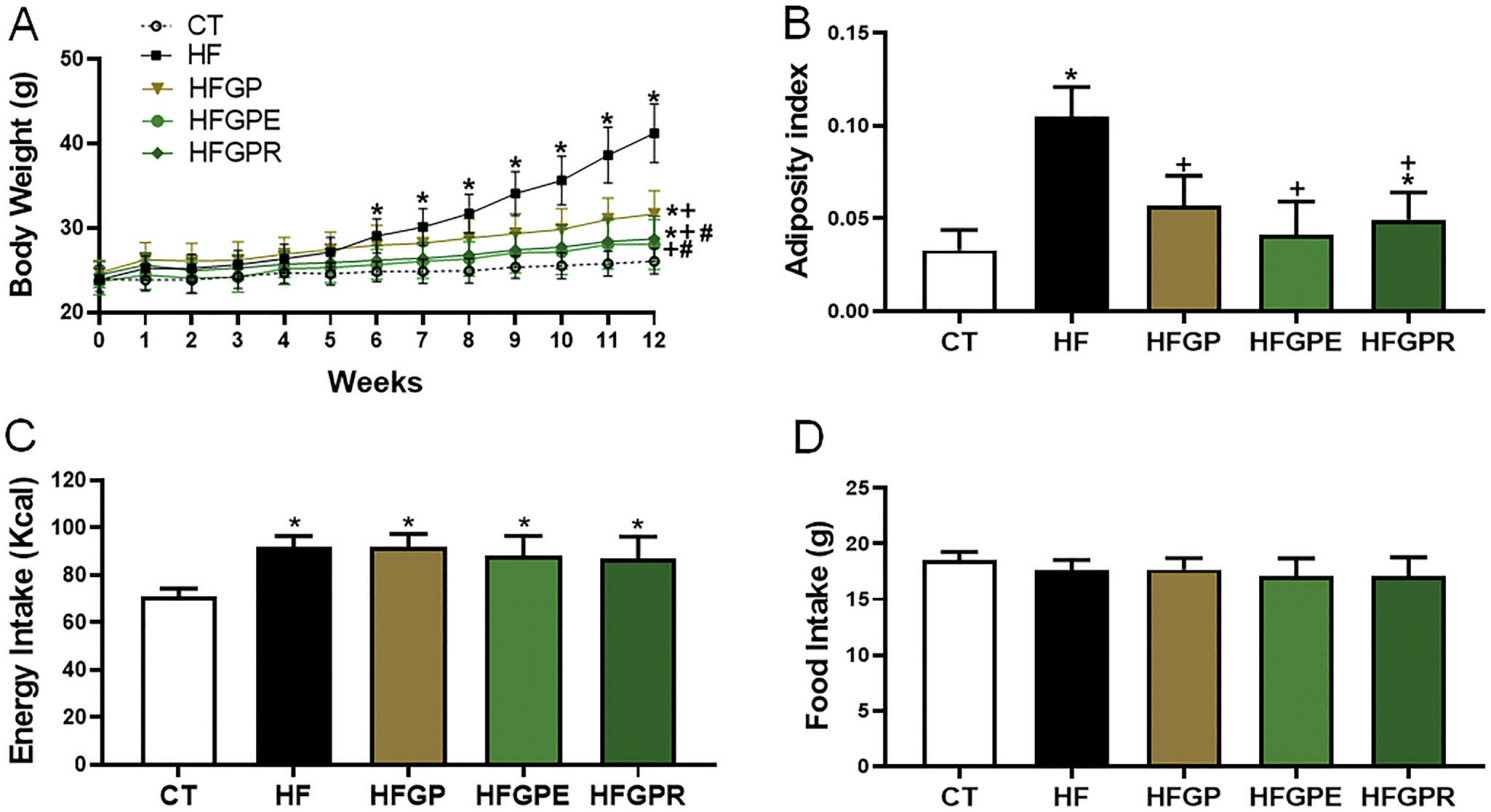
Effects of Brazilian green propolis supplementation (2%) on body weight (A), adipose index (B), energy intake (C), and food intake (D). Values are expressed as means ± SEM, n = 12–15 for all groups. ^*^Significantly different (*p* < 0.05) from the control group; ^+^significantly different (*p* < 0.05) from the HF group; ^#^significantly different (*p* < 0.05) from the HFGP group.

Body weight and the adiposity index were significantly increased (*p* < 0.05) in the HF group compared with the control group, confirming successful induction of obesity in this animal model (Figure 2A,B). Supplementation with GP and its residues resulted in a significant reduction (*p* < 0.05) in both body weight and adiposity index compared to the HF group, indicating a potential anti‐obesity effect.

As expected, no differences in food intake were observed among the experimental groups; however, the animals in the HF, HFGP, HFGPE, and HFGPR groups exhibited higher (*p* < 0.05) energy intake compared with the control group, as the HF diet provides greater caloric density than the standard diet (Figure 2C,D).

Based on the mean daily food consumption recorded throughout the experimental period and the mean body weight of each group, we estimated the actual propolis intake for each supplemented group. The HFGP group presented a mean food intake of 17.7 g/day and a mean body weight of 28.03 g, resulting in an estimated intake of approximately 12.630 mg/kg/day. Similarly, the HFGPE group consumed 17.11 g/day with a mean body weight of 25.8 g (∼13.264 mg/kg/day), and the HFGPR group consumed 17.14 g/day with a mean body weight of 26.4 g (∼12.985 mg/kg/day). The estimated bioactive intake was therefore consistent across all supplemented groups (range: ∼12.600–13.300 mg/kg/day), supporting the homogeneity of exposure throughout the 12‐week experimental period.

### 3.3 Biochemical Assays

As expected, initial glycemia did not differ between the groups (Table 3). After 10 weeks of HF diet‐induced obesity, all groups exhibited a significant increase in blood glucose levels (*p* < 0.05) compared with the control group, indicating a diet‐induced hyperglycemic state (Table 3). Supplementation with GP and its derivatives significantly reduced blood glucose levels (*p* < 0.05) in the HFGP, HFGPE, and HFGPR groups, indicating a potential hypoglycemic effect of GP (Table 3).

**TABLE 3:** Effects of Brazilian green propolis on glycemia, lipid profile, ALT, ALP, hepatic analysis, and oxidative stress in C57BL/6 mice.

Parameters	Control	HF	HFGP	HFGPE	HFGPR
Glycemia					
Initial glucose (mg/dL)	131.6 ± 2.8	138.3 ± 3.9	133.7 ± 3.6	139.8 ± 3.6	138.6 ± 3.4
Final glucose (mg/dL)	168.8 ± 2.6	204.1 ± 4.6^*^	185.2 ± 3.5^+^	178 ± 3.5^+^	175.7 ± 4.5^+^
Serum analysis					
TC (mg/dL)	61.4 ± 4.2	160.1 ± 7.8^*^	125.9 ± 6.4^*+^	136 ± 5.4^*+^	141.4 ± 5.5^*^
TG (mg/dL)	33.1 ± 3.1	54.8 ± 2.6^*^	36.1 ± 3.2^+^	40 ± 2.1^+^	31.5 ± 2^+^
LDL (mg/dL)	28.7 ± 3.2	94.8 ± 7.3^*^	61.1 ± 5.4^*+^	66.8 ± 7.2^*+^	60.9 ± 5.8^*+^
VLDL (mg/dL)	7.6 ± 0.8	10.6 ± 1.7^*^	7.5 ± 0.6^+^	8.1 ± 0.4^+^	6.7 ± 0.4^+^
HDL (mg/dL)	40.2 ± 5.7	57.4 ± 3.8	83 ± 5.4^*+^	81.1 ± 8.7^*+^	90.6 ± 4.1^*+^
ALT (U/L)	6.2 ± 1	16.2 ± 1^*^	10.9 ± 1.6^+^	8.6 ± 1.4^+^	6.7 ± 0.8^+^
ALP (U/L)	32.4 ± 3	52.9 ± 4.8^*^	30.9 ± 2.9^+^	29.6 ± 1.9^+^	30.7 ± 2.7^+^
Hepatic analysis					
TC (mg/dL)	7.5 ± 1.8	15.5 ± 1.5^*^	8.2 ± 1.8^+^	8 ± 1.1^+^	8.4 ± 1.1^+^
TG (mg/dL)	84.2 ± 4.8	512 ± 79.5^*^	197.8 ± 40.5^+^	110.3 ± 22.6^+^	90.2 ± 10.6^+^
MDA	0.13 ± 0.01	0.21 ± 0.03^*^	0.13 ± 0.007^+^	0.14 ± 0.006^+^	0.14 ± 0.009^+^
SOD	81.5 ± 5.3	49.6 ± 3.8^*^	91.2 ± 6.9^+^	72.3 ± 3.9^+^	72.6 ± 4.6^+^
Catalase	0.2 ± 0.02	0.14 ± 0.01^*^	0.26 ± 0.02^+^	0.25 ± 0.02^+^	0.24 ± 0.02^+^
GPx	0.015 ± 0.001	0.015 ± 0.001	0.014 ± 0.001	0.015 ± 0.001	0.015 ± 0.001

*Note*: Data are means ± SEM. One‐way ANOVA.

Abbreviations: ALP, alkaline phosphatase; ALT, alanine aminotransferase; GPx, glutathione peroxidase; HDL, high‐density lipoprotein; HF, high‐fat; HFGP, high‐fat green propolis; HFGPE, high‐fat green propolis extract; HFGPR, high‐fat green propolis residue; LDL, low‐density lipoprotein; SOD, superoxide dismutase; TBARS, thiobarbituric acid reactive substances; TC, total cholesterol; TG, triglycerides; VLDL, very low‐density lipoprotein.

^*^Significantly different from the control (*p* ≤ 0.05).

^+^Significantly different from the corresponding HF group (*p* ≤ 0.05).

At the 12th week, animals in the HF group exhibited marked alterations in the lipid profile, evidenced by significant (p < 0.05) increases in TC, TG, LDL, and VLDL levels (Table 3). Supplementation with GP and its derivatives effectively attenuated these metabolic disturbances. The significant increase in HDL cholesterol levels (*p* < 0.05) observed following supplementation suggests that GP exerts a beneficial hypolipidemic effect (Table 3).

Our results also demonstrated a significant increase (*p* < 0.05) in plasma ALT and ALP levels, key markers of hepatic function, in animals from the HF group compared with the control group, indicating substantial hepatic impairment induced by the HF diet (Table 3). Supplementation with GP and its extracts effectively improved liver function (*p* < 0.05) in the HFGP, HFGPE, and HFGPR groups, as evidenced by reductions in plasma levels of these hepatic enzymes (Table 3).

### 3.4 TC and TB Hepatic

Hepatic concentrations of cholesterol and triglycerides were elevated (*p* < 0.05) in the HF group relative to the control group (Table 3). Supplementation with GP and its extracts effectively reduced (*p* < 0.05) these lipid parameters in the HFGP, HFGPE, and HFGPR groups when compared with the HF group (Table 3).

### 3.5 Hepatic Oxidative Stress

The HF group exhibited elevated levels of oxidative damage markers, such as MDA (*p* < 0.05), along with reduced catalase antioxidant activity compared with the control group (Table 3). Supplementation with GP and its extracts effectively reduced oxidative damage (*p* < 0.05) and enhanced catalase activity, suggesting an antioxidant effect of GP that may contribute to the improvement of hepatic structural and functional alterations (Table 3). No significant differences in GPx activity were observed among the experimental groups (Table 3).

### 3.6 Hepatic Steatosis and Fibrosis

**FIGURE 3:**
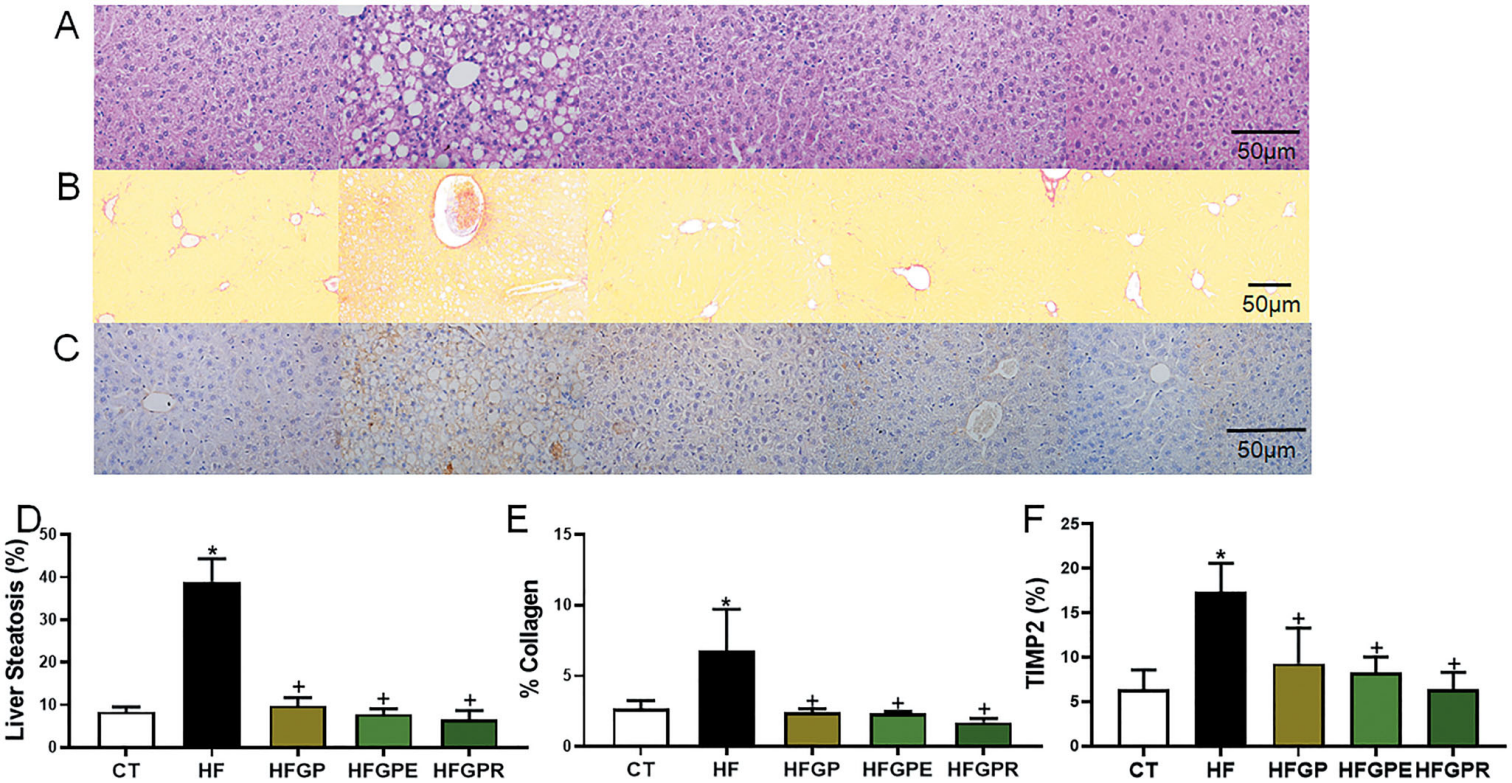
Effects of Brazilian green propolis supplementation (2%) on hepatic morphological changes, on hepatic fibrosis, and on TIMP‐2 immunostaining in the liver of C57BL/6 mice. Representative photomicrographs of the liver tissue stained with hematoxylin and eosin (100×, calibration bar = 50 µm) (A); representative photomicrographs of the liver tissue stained with Picrossirius Red (20×, calibration bar = 50 µm) (B); representative photomicrographs of the liver immunostaining with TIMP‐2 (C) (40×, calibration bar = 50 µm); hepatic steatosis (D); collagen surface (E); TIMP‐2 (F) of control, HF, HFGP, HFGPE, and HFGPR groups. Data are expressed as means ±SEM, *n* = 5 for all groups. ^*^Significantly different (*p* < 0.05) from the control group; ^+^significantly different (*p* < 0.05) from the HF group.

Histological examination of liver sections revealed a marked accumulation of lipid droplets in the hepatocytes of the HF group compared with the control group (*p* < 0.05), indicating hepatic steatosis (Figure 3A,D). Conversely, all groups supplemented with GP exhibited a significant reduction (*p* < 0.05) in hepatic lipid deposition compared with the HF group. This finding is further supported by the elevated plasma triglyceride levels (p < 0.05) observed in the HF animals (Table 3). In contrast, supplementation with GP significantly lowered these levels (*p* < 0.05), highlighting its beneficial role in mitigating hepatic steatosis.

Exposure to the HF diet led to increased collagen deposition in the livers of HF animals (*p* < 0.05) compared with the control group, indicating the development of hepatic fibrosis (Figure 3B,E). Supplementation with GP and its extracts effectively attenuated (*p* < 0.05) this alteration, reinforcing the hepatoprotective effect of GP in animals subjected to the HF diet (Figure 3B,E).

Animals fed an HF diet exhibited increased hepatic TIMP‐2 content (*p* < 0.05), a key regulator of extracellular matrix (ECM) remodeling that plays an important role in hepatic fibrogenesis (Figure 3C,F). Supplementation with Brazilian GP, either as the hydroalcoholic extract or the residue‐derived extract, significantly reduced TIMP‐2 immunostaining, suggesting a marked antifibrotic effect (Figure 3C,F).

### 3.7 Hepatic Molecular Markers Associated With Lipogenesis and Inflammation

**FIGURE 4:**
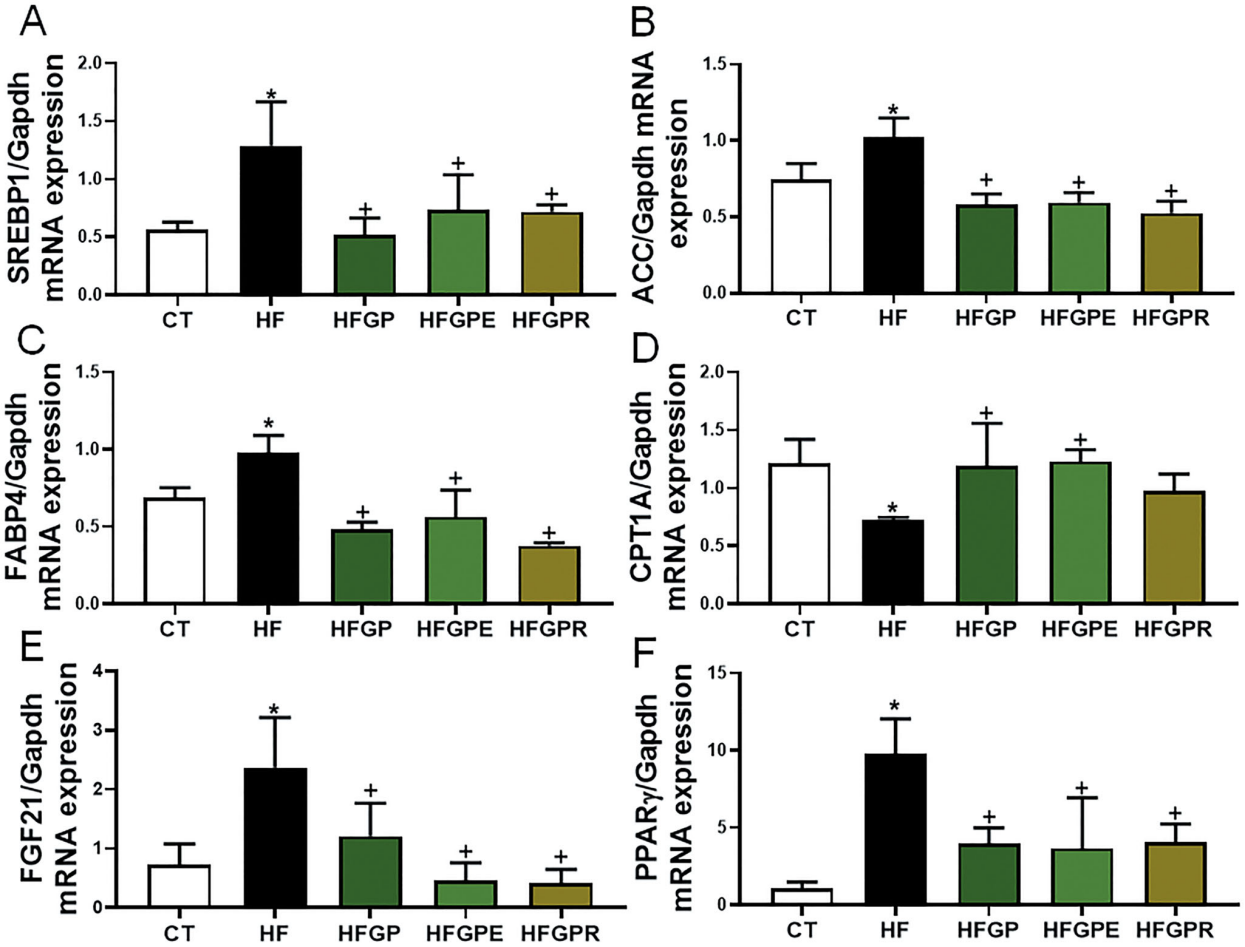
Effects of Brazilian green propolis supplementation (2%) on the gene expression of lipogenesis markers SREBP1 (A), ACC (B), FABP4 (C), CPT1A (D), FGF21 (E), and PPARγ (F), relative to Gapdh mRNA levels, were evaluated in liver tissue. Data are expressed as means ± SEM, *n* = 5 for all groups. ^*^Significantly different (*p* < 0.05) from the control group; ^+^significantly different (*p* < 0.05) from the HF group.

PCR analysis revealed that the hepatic expression of *Srebp1, Acc, Fabp4*, *Cpt1a, Fgf21*, and *Ppargamma* was significantly upregulated (*p* < 0.05) in animals from the HF group compared with the control group, indicating enhanced lipogenic and metabolic stress responses induced by the HF diet (Figure 4A–F). Supplementation with GP effectively improved (*p* < 0.05) the expression of these genes, demonstrating its regulatory effect on hepatic lipid metabolism and its potential to mitigate diet‐induced metabolic disturbances (Figure 4A–F).

**FIGURE 5:**
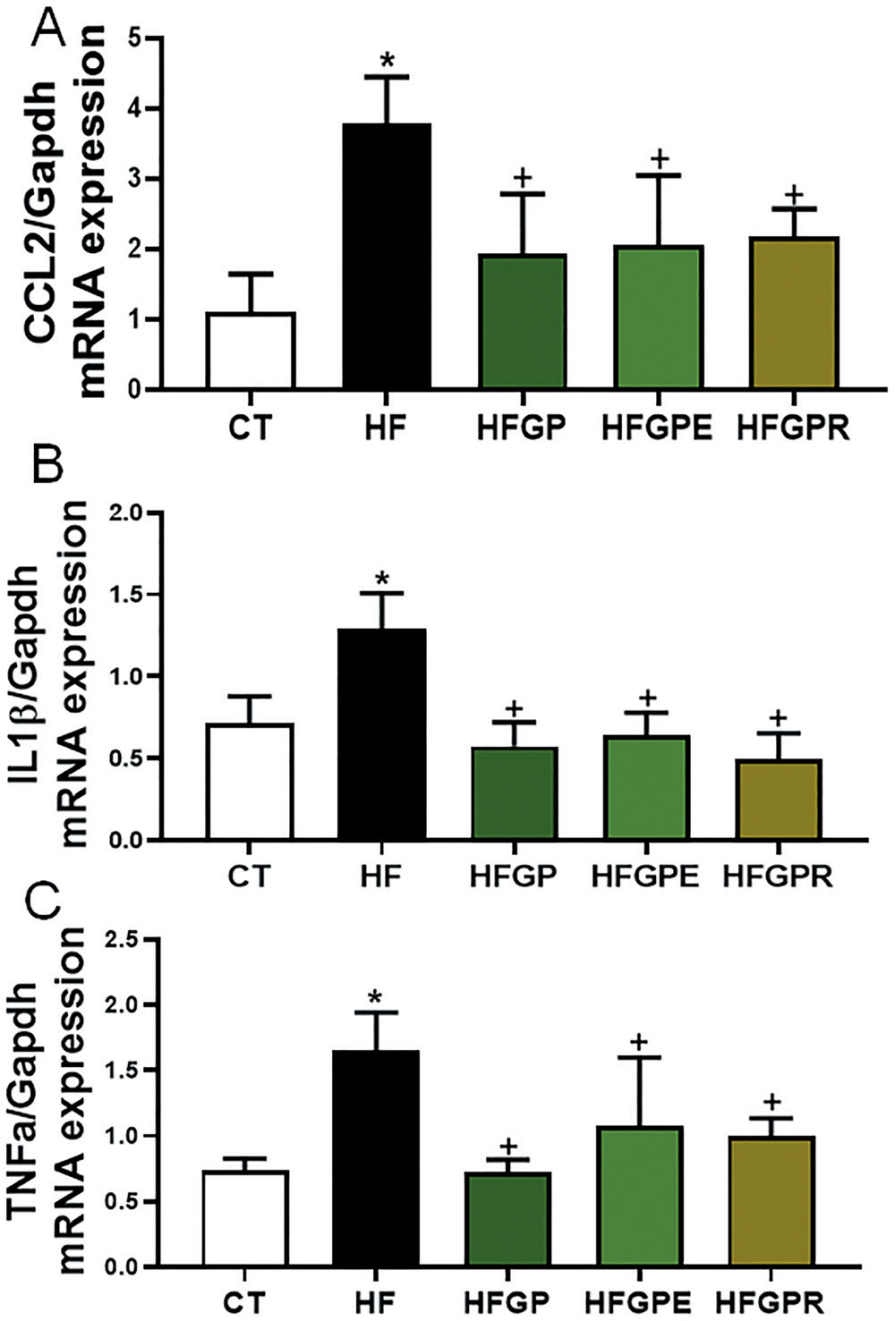
Effects of Brazilian green propolis supplementation (2%) on the gene expression of the inflammatory markers CCL2 (A), IL1β (B), and TNFα (C), relative to Gapdh mRNA levels, were evaluated in liver tissue. Data are expressed as means ± SEM, *n* = 5 for all groups. ^*^Significantly different (*p* < 0.05) from the control group; ^+^significantly different (*p* < 0.05) from the HF group.

The hepatic gene expression analysis demonstrated a pronounced upregulation (*p* < 0.05) of *Ccl2, Il1b*, and *Tnfα* in the HF group compared with the control group, indicating activation of inflammatory pathways induced by the HF diet (Figure 5A–C). Supplementation with GP significantly downregulated (*p* < 0.05) the expression of these pro‐inflammatory markers, highlighting its potent anti‐inflammatory and hepatoprotective effects (Figure 5A–C).

## 4 Discussion

The present study evaluated the effects of Brazilian GP and two derived extracts on metabolic and hepatic alterations in an experimental obesity model. These effects may be associated, at least in part, with the complex chemical composition identified by HPLC‐DAD. Notably, this study advances current knowledge by exploring the impact of GP on hepatic lipogenesis. This area remains poorly understood and can be addressed by proposing a sustainable strategy based on the reuse of bioactive extraction residues.

Artepillin C was identified as a major constituent in both extracts (22.3% in GPE and 23.4% in GPR), alongside other prenylated compounds, including drupanin and baccharin. As a key biomarker of GP, APC exhibits well‐established antioxidant and anti‐inflammatory properties (Cai et al. 2020) and may contribute to modulating hepatic metabolism and adipocyte dysfunction (Okamura et al. 2022). The identification of these bioactive compounds in the residue‐derived extract further supports its potential as a sustainable source of natural compounds for the management of metabolic disorders such as MASLD.

Using an HF diet (HFD)‐induced obesity model (Tavares et al. 2020; De Moraes Arnoso et al. 2022), supplementation with GP and the evaluated propolis‐derived preparations significantly reduced body weight gain and adiposity. Although previous studies reported inconsistent effects on body weight (Okamura et al. 2022; Kanazashi et al. 2023; De Miranda et al. 2023), the present findings reinforce the beneficial effects of Brazilian GP preparations against obesity‐induced metabolic alterations.

Metabolic disturbances, including hyperglycemia and dyslipidemia, are key drivers of hepatic steatosis and MASLD progression (D. Liu et al. 2025). In this study, the evaluated propolis preparations improved lipid and glucose homeostasis, reduced hepatic lipid accumulation, and attenuated steatosis. These findings align with evidence showing the modulation of cholesterol metabolism, the inhibition of 3‐Hydroxy‐3‐Methylglutaryl‐Coenzyme A (HMG‐CoA reductase, and improved metabolic profiles following propolis supplementation (Okamura et al. 2022; De Miranda et al. 2023).

Additionally, reduced serum ALT and ALP levels are consistent with hepatoprotective effects (Xuan et al. 2024; Nikbaf‐Shandiz et al. 2022) and may be associated with antioxidant and anti‐inflammatory activities previously reported for propolis and its constituents (Li et al. 2024). Histological and biochemical improvements further confirmed attenuation of hepatic steatosis and fibrosis, consistent with previous reports involving propolis bioactives such as APC (Okamura et al. 2022; De Miranda et al. 2023; Nazari‐Bonab et al. 2024).

Oxidative stress plays a central role in the progression of steatosis (Martín‐Fernández et al. 2022; Svobodová et al. 2025). HFD‐fed mice exhibited increased lipid peroxidation (MDA) and reduced antioxidant enzyme activity (SOD, catalase), whereas supplementation restored redox balance. These effects are consistent with previous studies suggesting the involvement of cytoprotective pathways (e.g., Nrf2/HO‐1, PI3K/Akt) and radical‐scavenging properties of polyphenols (Kanazashi et al. 2023; Nazari‐Bonab et al. 2024; Zhu et al. 2023).

At the molecular level, supplementation with the propolis preparations modulated key regulators of lipid metabolism. Specifically, it downregulated lipogenic genes (SREBP1, acetyl‐CoA carboxylase [ACC], fatty acid binding protein 4 [FABP4]) and upregulated carnitine palmitoyltransferase 1A (CPT1A), promoting fatty acid oxidation, consistent with previous studies (Zhu et al. 2023; Saleh Al‐maamari et al. 2021; C. Liu et al. 2024; Mahboob et al. 2023). These findings are consistent with AMP‐Activated Protein Kinase (AMPK)‐mediated regulation of lipid metabolism, which has been reported to reduce malonyl‐CoA levels and enhance mitochondrial β‐oxidation (Mahboob et al. 2023); however, direct measurement of AMPK activation was not performed in the present study.

Furthermore, modulation of peroxisome proliferator‐activated receptor gamma (PPARγ) and fibroblast growth factor 21 (FGF21) suggests coordinated regulation of lipid and energy metabolism. While PPARγ is linked to lipid accumulation, partial modulation by the propolis preparations may improve insulin sensitivity (M. Wang et al. 2023; Chen et al. 2022), whereas the observed modulation of FGF21 expression suggests a potential role in enhanced lipid oxidation and metabolic adaptation, though the downstream effects of this regulation were not directly evaluated (Zhu et al. 2023; Saleh Al‐maamari et al. 2021; C. Liu et al. 2024). Downregulation of these markers in the present study is consistent with improved metabolic homeostasis, although further mechanistic studies are needed to establish causal relationships.

Inflammation is a key driver of progression from steatosis to steatohepatitis. HFD increased hepatic expression of proinflammatory mediators (C‐C motif chemokine ligand 2 [CCL2], TNF‐α, IL1B), whereas propolis supplementation significantly attenuated these markers, consistent with its anti‐inflammatory properties (Martins‐Gomes et al. 2024; Zulhendri et al. 2022). Reduced CCL2 expression may limit macrophage recruitment and inflammatory amplification (Zulhendri et al. 2022; Zamarrenho et al. 2023).

Additionally, increased TIMP‐2 expression in HFD animals suggests early fibrotic remodeling (Rayginia et al. 2025). Supplementation with the evaluated propolis preparations reduced TIMP‐2 levels, suggesting potential antifibrotic effects (Martín‐Fernández et al. 2022; Rayginia et al. 2025). However, the involvement of the MMP/TIMP balance was not directly investigated and therefore remains speculative.

Collectively, these findings demonstrate that treatment with Brazilian GP and the evaluated propolis‐derived preparations was associated with coordinated metabolic, antioxidant, anti‐inflammatory, and antifibrotic effects that mitigated obesity‐associated hepatic steatosis. The identification of biological activity in the residue‐derived preparation supports further investigation into the valorization of propolis processing by‐products as a potential sustainable source of bioactive compounds. However, additional studies using chemically standardized preparations are required to establish the relative biological potency of each material (Zulhendri et al. 2022; Bahari et al. 2025).

The present study has several limitations that should be acknowledged. First, the experimental model was restricted to male C57BL/6 mice. Although this model is widely validated for studies of diet‐induced obesity and metabolic dysfunction, the findings cannot be directly extrapolated to females, other age groups, different species, or human clinical settings. Second, although the gene expression and biochemical findings are consistent with modulation of key pathways involved in lipogenesis, oxidative stress, and inflammation, direct confirmation of pathway activation through protein expression or phosphorylation analyses (e.g., AMPK, Nrf2, and PPARγ) was not performed. Therefore, the mechanistic interpretations proposed herein should be considered preliminary.

Another important limitation concerns the chemical standardization of the propolis preparations. Although the major constituents were identified by HPLC‐DAD and the total phenolic and flavonoid contents were determined, the preparations were incorporated into the diets on a gravimetric basis (2%, w/w) rather than being standardized according to their ethanol‐soluble extractive content or specific chemical markers. Furthermore, the chromatographic data were expressed as relative peak area percentages and were intended primarily to characterize the compositional profiles of the different preparations rather than to provide absolute quantification of individual compounds. Consequently, the absolute amounts of ethanol‐soluble bioactive constituents administered may have differed among treatments, particularly between raw propolis and the extract‐based preparations. Therefore, any differences observed among the preparations may have been influenced, at least in part, by differences in the amount of ethanol‐soluble compounds delivered rather than exclusively reflecting intrinsic biological differences. Future studies should employ chemically standardized preparations to enable direct comparisons of biological potency and to identify the specific constituents responsible for the observed biological activities.

Finally, all supplemented groups received a fixed dietary concentration (2%, w/w), and no dose–response study was performed. Future investigations should evaluate different doses to establish optimal bioactive intake levels, determine long‐term safety, and further explore the translational potential of residue‐derived extracts for the management of metabolic diseases.

Despite these limitations, the present study provides novel evidence supporting the metabolic and hepatoprotective potential of Brazilian GP and propolis‐derived preparations in an experimental model of obesity‐induced MASLD. The findings demonstrate that the hydroalcoholic extract obtained from residues generated during essential oil extraction exhibits biologically relevant activity, supporting the concept of residue valorization as a sustainable strategy for the development of propolis‐derived products. In addition, the modulation of key molecular regulators involved in lipogenesis, oxidative stress, and inflammation advances the current understanding of the mechanisms underlying the beneficial effects of Brazilian GP in metabolic liver disease.

From a technological perspective, the present work should be regarded as a proof‐of‐concept for the integrated utilization of Brazilian GP. Although the commercial production of Brazilian GP essential oil is currently limited, its expanding potential applications in the pharmaceutical, cosmetic, and food industries may stimulate future industrial production. Under such a scenario, the recovery of bioactive compounds from the solid residue generated during essential oil extraction could represent an economically and environmentally attractive strategy. Rather than being discarded, this material may serve as a valuable source of bioactive compounds, contributing to a circular production chain that maximizes raw material utilization, minimizes waste generation, and adds value to propolis‐derived products in accordance with the principles of green chemistry and the circular bioeconomy.

## 5 Conclusions

In conclusion, the present preclinical study demonstrated that animals fed an HF diet developed marked metabolic and hepatic disturbances consistent with obesity‐induced steatosis. Supplementation with Brazilian GP, the conventional hydroalcoholic extract (GPE), and the hydroalcoholic extract obtained from the residue remaining after essential oil extraction (GPR) effectively ameliorated several metabolic, biochemical, oxidative, inflammatory, molecular, and histopathological alterations induced by the HF diet. Although the three preparations were compared on a gravimetric basis (2%, w/w) rather than according to equivalent concentrations of ethanol‐soluble bioactive constituents, all three propolis‐derived products exhibited biological activity under the experimental conditions employed.

Notably, GPR produced beneficial effects comparable to those of the conventional hydroalcoholic extract (GPE), despite being obtained from the residue remaining after essential oil extraction, supporting its potential as a value‐added product within a sustainable strategy for the utilization of Brazilian GP. These findings reinforce the concept that residues generated during essential oil extraction may represent an important source of bioactive compounds with metabolic benefits, contributing to waste valorization and supporting the principles of green chemistry and the circular bioeconomy.

Overall, these findings suggest that Brazilian GP and its derivatives may represent promising adjunct strategies for mitigating obesity‐associated metabolic disturbances. However, because the treatments were not chemically standardized according to their ethanol‐soluble extractive content or specific bioactive constituents, the present results should not be interpreted as evidence of equivalent biological potency among GP, GPE, and GPR. Further preclinical studies employing chemically standardized preparations, dose–response analyses, and subsequent clinical investigations are warranted before translational conclusions can be drawn.

**Nomenclature**
ACC: acetyl‐CoA carboxylase
ALT: alanine aminotransferase
ALP: alkaline phosphatase
APC: artepillin C
CCL2: C‐C motif chemokine ligand 2
CPT1A: carnitine palmitoyltransferase 1A
DPPH: 2,2‐diphenyl‐1‐picrylhydrazyl
FABP4: fatty acid binding protein 4
FGF21: fibroblast growth factor 21
FRAP: ferric reducing antioxidant power
GAE: gallic acid equivalents
GP: green propolis
GPE: green propolis hydroalcoholic extract
GPR: green propolis residue
GPx: glutathione peroxidase
HCC: hepatocellular carcinoma
HDL: high‐density lipoprotein
HF: high‐fat diet
HFGP: HF diet supplemented with green propolis 2%
HFGPE: HF supplemented with green propolis extract 2%
HFGPR: HF supplemented with green propolis residue 2%
HPLC: high‐performance liquid chromatography
LDL: low‐density lipoprotein
MASH: metabolic dysfunction‐associated steatohepatitis
MASLD: metabolic dysfunction‐associated liver disease
MDA: malondialdehyde
PPARγ: peroxisome proliferator‐activated receptor gamma
QE: quercetin equivalents
SOD: superoxide dismutase
SREBP1: sterol regulatory element‐binding protein 1
T2DM: type 2 diabetes mellitus
TBARS: thiobarbituric acid reactive substances
TC: total cholesterol
TF: total flavonoid content
TFen: total phenolic content
TG: triglycerides
TNFα: tumor necrosis factor alpha
VLDL: very‐low‐density lipoprotein

## Author Contributions

**Caroline Alves de Araújo**: writing – review and editing, methodology, conceptualization, investigation, visualization, formal analysis, data curation. **Giovana Dias Ramundo**: formal analysis, investigation, visualization, data curation, methodology. **Beatriz Teixeira dos Santos**: methodology, investigation, formal analysis, data curation, visualization. **Dafne Lopes Beserra Silva**: formal analysis, writing – review and editing, investigation, data curation, visualization. **Graziele Freitas de Bem**: conceptualization, writing – review and editing, investigation. **Dayane Teixeira Ognibene**: conceptualization, writing – review and editing, investigation. **Angela Castro Resende**: writing – review and editing, conceptualization, investigation, validation, formal analysis, data curation. **Brenda Akemi Nagagata**: methodology, writing – review and editing, visualization, conceptualization, investigation, formal analysis, data curation. **Isabela Macedo Lopes Vasques‐Monteiro**: conceptualization, investigation, methodology, visualization, writing – review and editing, formal analysis, data curation. **Julio Beltrame Daleprane**: methodology, conceptualization, investigation, visualization, writing – review and editing, formal analysis, data curation. **Debora Baptista Pereira**: methodology, conceptualization, investigation, writing – review and editing, visualization, formal analysis, data curation. **Douglas Siqueira de Almeida Chaves**: conceptualization, investigation, writing – review and editing, visualization, methodology, data curation, formal analysis. **Cristiane Aguiar da Costa**: conceptualization, investigation, funding acquisition, methodology, visualization, writing – review and editing, formal analysis, project administration, supervision, data curation, writing – original draft.

## Funding

This work was supported by the National Council for the Development of Science and Technology (CNPq, No. 303023/2022‐8); the Rio de Janeiro State Research Agency (FAPERJ, No. E‐26/211.195/2021; No. E‐26/200.196/2023), and the Coordination for the Improvement of Higher Education Personnel.

## Conflicts of Interest

The authors declare no conflicts of interest.

## Data Availability

The data underlying this article are available in the article.
